# Review of Two Siblings with Werner's Syndrome: A Case Report

**DOI:** 10.1155/2009/138312

**Published:** 2010-02-07

**Authors:** Murat Sert, Koray Fakioglu, Tamer Tetiker

**Affiliations:** Division of Endocrinology, Department of Internal Medicine, Medical Faculty, Cukurova University, 01330 Adana, Turkey

## Abstract

We report the clinical course of two siblings with Werner's syndrome (WS) who were diagnosed and followed at our clinics for 12 years. Initial diagnosis of the first sibling (sister) was at age 20, the second (brother) at 16. At the initial diagnosis, the sister had amenorrhea, muscle atrophy at arms and legs, diabetes mellitus (DM), short stature, bilateral cataracts, genital hypoplasia, osteoporosis, and gray hair. During 12 years follow-up period, high-pitched voice, hepatosteatosis, renal parenchymal disease, and urethral obstruction developed. Regarding the brother, DM, cataracts and genital hypoplasia were observed at the initial diagnosis. During the 12 years follow-up period, gray hair, high-pitched voice, steatohepatosis, and osteoporosis developed.

## 1. Introduction

Werner's syndrome is an autosomal recessive disorder affecting the connective tissue of the whole body. It is also known as progeria adultorum and pangeria. Its clinical manifestations are short stature, scleroderma-like skin alterations, cataracts, and premature aging of the face. Werner's syndrome was first described by Otto Werner at 1904. At that time, there were observed juvenile cataracts, scleroderma-like skin alterations, short stature, premature aging of the face, gray hair, and genital hypoplasia in 4 siblings [1]. At 1934 Oppenheimer and Kugel [2] described the additional endocrinological abnormalities such as osteoporosis and hyperglycemia (see Table 1). Between 1916 and 2002, 1300 cases were reported worldwide. Here, we reported two siblings with WS who diagnosed and followed at our clinic and clinical progression of WS since the initial diagnosis.

## 2. Case 1 (see Figure 1)

As an outpatient female patient with 20 years old was admitted to our university endocrinology clinic with the complaining of thinning in her both wrists in 1997. In her past medical history, any disorder related to current disease was present in her parents. She had two brothers. One of her two brothers (second case) had DM, cataracts, genital hypoplasia, the other was normal. On her first examination, she had a short stature (143 cm) and her extremities were thin. Her nose had a bird-like appearance. After menarche she had menstruation regularly for 3 years but later on it was interrupted. She had bilateral cataracts. An abdominal ultrasonography showed genital hypoplasia. She had osteopenia revealed by DEXA (T-score: −1,55). FSH, LH, and estradiol levels were higher than normal ranges. She had gray hair. By 2004, an examination due to high-pitched voice revealed that right vocal cord was paralytic, and left vocal cord movement was normal. By 2007, a clinical evaluation disclosed that hepatosteatosis and grade 1 renal parenchymal disease. By 2008, she had an operation due to urethral obstruction and difficulty in voiding.

## 3. Case 2 (see Figure 2)

He was seen and diagnosed at age 16 during the screening of his sister's disease (Case 1). On admission his pathological findings were cataracts, DM, short stature (159 cm), and genital hypoplasia (micropenis and right cryptorchidism). By 2002, a steatohepatosis was found by liver biopsy which was performed for the persisting high liver transaminases. By 2007 gray hair and high-pitched voice developed. On examination, bilateral vocal cords were functioning normally and a gap was present. He had osteopenia (T-score: −1.4) disclosed with a DEXA examination in 2007. His serum FSH and LH levels were higher than normal ranges, while his testosterone level was below the normal ranges (hypergonadotropic hypogonadism).

## 4. Discussion

Werner's syndrome results from a mutation at WS gene belonging to Rec Q helicase family [4]. Its estimated incidence is 1 case in 1 million individuals. It is more prevalent in Japan and Sardinia. Mean survival time is 46 years and death is mostly due to atherosclerosis and malign tumors. Mesenchymal sarcoma is seen 10 times more. Other malignancies with elevated incidences are malign melanoma, thyroid cancer, osteosarcoma, and soft tissue sarcoma. Immunological abnormalities [5] and DNA abnormalities [6] are found to be associated with development of malignancies. At diagnosis age is usually 37 and syndrome occurs mostly after puberty. The most important theory explaining the development of the disease is abnormal metabolism of connective tissue as shown by pathological and biochemical studies since at WS, abnormal mucopolysaccharides and fibroblast are found [7, 8]. In a study by Ariyoshi et al., it was demonstrated that a strain of WS fibroblast cells shows abnormal karyotypes characterized by several complex translocations and 50-fold more frequency of abnormal metaphases including dicentric choromosomes without fragments than normal cells when examined at a similar culture stage. Furthermore, telomere fluorescence in situ hybridization indicated that the abnormal signals, extra telomere signal and loss of telomere signal, emerge two- to threefold more frequently in WS cells than in normal cells. Taken together, these results indicate that choromosome instability including dysfunction of telomere maintenance is more prominenet in WS cells than in normal cells. In addition, it was reported that the accumulation of DNA double-strand breaks (DSBs) at the G(1) phase, including those at telomeres foci; was accelerated in WS cells even at a low senescence level. These results indicate that WS cells are prone to accumulate DSBs spontaneously due to a defect of WRN gene, which leads to increased choromosome instability that could activate checkpoints, resulting in accelerated senescence [3]. In the differential diagnosis of WS, progeria, acrogeria, Rothmund-Thomson syndrome (RTS), and Cockayne syndrome (CS) included. Progeria is a rare combination of dwarfism and premature aging. The probable cause is a mutation in the lamin located in the nuclear matrix [9]. Increase in the blood hyaluronic acid levels is responsible for sclerodermatous changes and cardiovascular abnormalities. The affected children are normal at birth and grow normally till about the end of the first year, when both normal growth and gain in weight slow down. At the end of the first decade, the size attained is that of a normal child of three years of age. Loss of hairs and subcutaneous fat along with sclerodermatous changes gives rise to characteristic plucked bird appearance at about 6–12 months of age. Scalp hair and eyelashes are progressively lost with increased prominence of scalp veins. Acrogeria is a progeroid syndrome of premature aging of the skin without the involvement of internal organs seen in progeria. It is seen mainly in females and in the form of sporadic cases. Familial cases are also seen. Acro-osteolysis of the distal phalanges, delayed cranial suture closure with wormian bones, linear lucent defects of the metaphyses, and antegonial notching of the mandible are the predominant skeletal features of the disorder [10]. Rothmund-Thomson syndrome is a hereditary and familial disease characterized by short stature, cataracts, pigmentation of skin, baldness, abnormalities of bones, nails, and teeth. Cockayne syndrome spans a spectrum that includes CS type 1, the classic form, CS type 2, a more severe form with symptoms present at birth (i.e., cerebro-oculofacial-skeletal syndrome, Pena-Shokeir type 2 syndrome), CS type 3, a milder form, and xeroderma pigmentosa-cockayne syndrome. Cockayne syndrome type 1 and type 2 are autosomal recessive disorders that feature growth deficiency, premature aging, and pigmentary retinal degeneration along with a complement of other clinical findings. Type 1 presents at birth; whereas type 2 appears during early childhood. Fatality usually occurs in early adolescence, but some patients survive until early adulthood. 

 Considering the presented two siblings with WS, we relatively diagnosed at the early ages of the patients with respect to the literature (20 yr versus 37 yr). Our diagnosis was based on the clinical findings and family history. Unfortunately, we could not perform genetic study due to our laboratory condition. We would like to contribute to the literature related with our clinical observation during the 12-yr follow-up. Generally, they were presented with premature grey hair, extremity pains, and thinning especially in the wrists with atrophic skin changes and hypogonadism; during the 12-yr follow-up, vocal cord paralysis with high pitched voice, osteopenia, and connective tissue problems developed. They are still under our clinical observation.

## Figures and Tables

**Figure 1:**
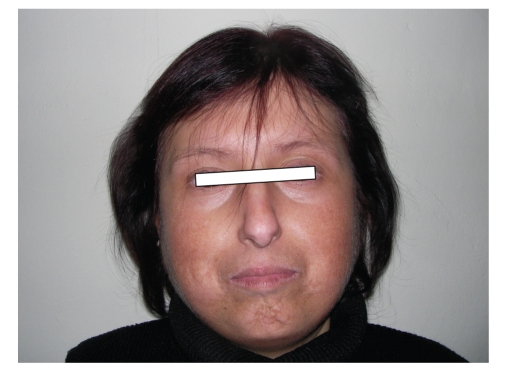
Case 1.

**Figure 2:**
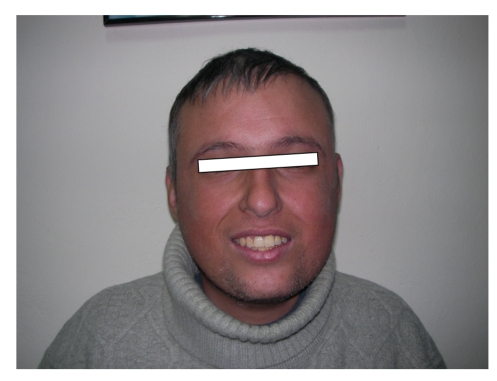
Case 2.

**Table 1:** Characteristic features of Werner's syndrome.

Diagnostic criteria by Irwin and Ward [3]	Incidence of features reported (%) in Japanese cases (*n* = 411)
Characteristic habitus and stature	
Short stature (from adolescence)	86.6
Slender extremities with stocky trunk	86.3
Beak-shaped nose	75.7
Premature senility	
Premature grey hair	86.1
Premature baldness	70.0
Atrophic skin	85.4
Weak and high-pitched voice	76.1
Arteriosclerosis	54.0
Juvenile cataracts	94.8
Scleroderma-like changes	
Atrophic skin and subcutaneous tissues	86.3
Circumscribed hyperkeratosis	70.5
Ulcers over the malleoli of the ankles, Achilles tendon, heels, and toes	69.5
Other manifestations	
Tendency to diabetes mellitus	67.2
Hypogonadism	64.2
Osteoporosis	54.7
Localised calcification	57.4
Tendency to occur in siblings	48.7

Data obtained by the authors' search of MEDLINE and Japanese databases in the years from 1916 to 2002.
